# Supplementary material for: Miyeokgui (Undaria pinnatifida Sporophyll) Characteristic under Different Relative Humidity: Microbial Safety, Antioxidant Activity, Ascorbic Acid, Fucoxanthin, α-/β-/γ-Tocopherol Contents
Source: Foods. 2023 Jun 11;12(12):2342. doi: 10.3390/foods12122342 (PMC10296908; doi:10.3390/foods12122342)
Supplement: Supplementary file 1 [file foods-12-02342-s001.zip › foods-2420541-supplementary.pdf]

Figure S1. Moisture sorption isotherm curve of UPS powder as affected by different RHs storage.

Figure S2. Appearance of UPS powder as affected by different RHs.

Figure S1.

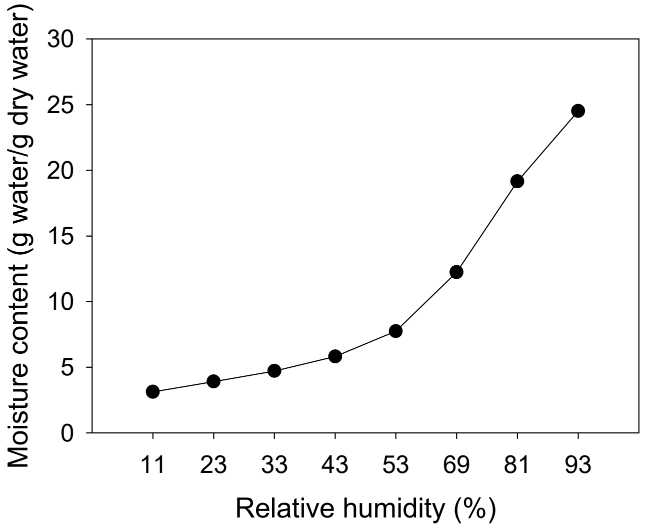

Moisture content (g water/g dry water)
30
25
20
15
10
5
0
11
23
33
43
53
69
81
93
Relative humidity (%)

Figure S2.

Storage time (week)	% Relative humidity							
	11	23	33	43	53	69	81	93
2	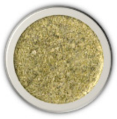	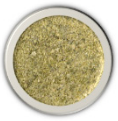	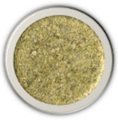	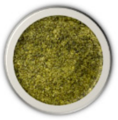	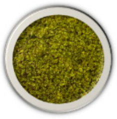	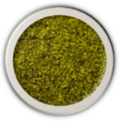	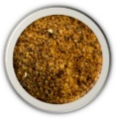	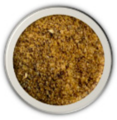
3	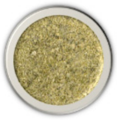	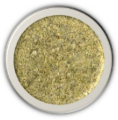	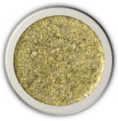	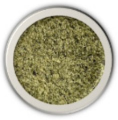	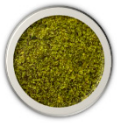	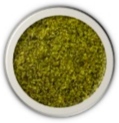	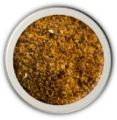	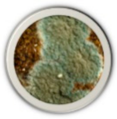
4	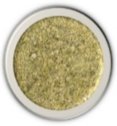	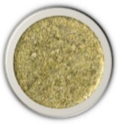	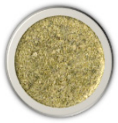	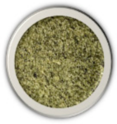	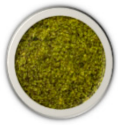	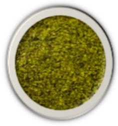	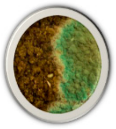	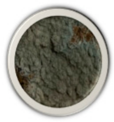
